# No-shows in ambulatory clinics and non-utilized appointments for elective operations in selected surgical departments at a tertiary hospital in Israel

**DOI:** 10.1186/s13584-019-0333-5

**Published:** 2019-07-30

**Authors:** Lior Cohen-Yatziv, Matan Joel Cohen, Jonathan Halevy, Ehud Kaliner

**Affiliations:** 1 Hadassah-Hebrew University Medical Center, Jerusalem, Israel; 2 Clalit Health Services, affiliated with the Hebrew University Faculty of Medicine, Yigal Alon 1, Beit-Shemesh, Jerusalem district Israel; 3 Shaare Zedek Medical Center, Shmuel Bait St 12, 9103102 Jerusalem, Israel; 4 Israel Ministry of Health - Ministry of Health, Yermiyahu St 39, 9101002 Jerusalem, Israel

**Keywords:** No-show, Ambulatory care, Operating room usage, Missed appointments, Post-operative meeting, Surgery cancellation

## Abstract

**Background:**

The phenomenon of a patient missing a medical appointment without notification is called a “no-show”. In contrast, “non-utilized appointments” are a broader phenomenon including all appointments that didn’t occur as registered – whether due to actions taken by providers or patients. Both no-shows and non-utilized appointments can lead to reduced quality of care, loss in productivity, financial losses and impaired patient outcomes.

**Methods:**

The study was carried out between August 2016 and January 2017 in the ENT, Orthopedics and General Surgery Departments of the Jerusalem-based Shaare Zedek Medical Center. The study team sought to examine the reasons for non-utilized appointments in elective operations. The study team also interviewed no-show ambulatory care patients regarding the causes of the no-show and reviewed medical records of no-show patients to determine the nature of the missed appointments.

**Results:**

The rate of non-utilization of appointments for elective operations was 6%. The leading reasons for non-utilization of these appointments were: patient health issues, patient surgery postponement and surgery schedule overload (together accounting for 52% of cases and 72% of known reasons). The no-show rate for ambulatory clinic appointments was approximately 15%. The leading reasons for ambulatory clinic no-shows were: administrative issues, illness and forgetfulness (together accounting for 58% of all reasons). The leading types of appointments missed were:post-operation follow-ups and chronic illness follow-up (together accounting for 46% of cases and 63% of known reasons).

**Conclusions:**

In this study, the non-utilized appointment rate for elective operations was found to be lower than those noted in the medical literature, while the no-show rate for ambulatory visits was found to be similar to that found in the literature. There is room to question the necessity of certain types of postoperative follow-up appointments since they are at “high risk” for no-show. One promising way to reduce the no-show rate would involve improving the hospital’s information and computing systems in order to identify patients who are susceptible to a no-show incident.

## Background

An event during which a patient does not arrive for a scheduled appointment is defined as a “no-show”. This phenomenon is common in primary and secondary care [1] and can be seen in a variety of population groups [2]. No-show events have been shown to be associated with poor results in the treatment of both sporadic medical appointments, and in cases of chronic disease management [3].

It is estimated that between 10 and 30% of scheduled hospital medical ambulatory appointments end-up as no-show events [4], and this rate could even reach 50% in primary care [5]. It appears that only a small percentage of patients (between 10 and 20%) is responsible for a significant proportion of the no-show events - approximately 30 to 40% of them [2].

No-show events can compromise patient health; for example, they can be a missed opportunity for medical diagnosis [3]. A high incidence of no-show events reduces the availability of appointments and increases the waiting time for appointments [2]. Consequently, many other patients who are waiting for an appointment may become displeased and in some cases the quality of their care could be adversely affected.

No-shows can also be costly. In a nationwide study in Britain, the cost of no-show events during 2004 was estimated at £790 million [6], which constitutes 1% of national health expenditures in the UK in that year [7].

In contrast, non-utilized appointments are a broader phenomenon than “no shows”. They include all appointments that did not occur as registered, whether due to patient or provider actions. They include both no-show events and other events in which planned appointments did not take place and resources were left un-utilized. The latter included cases in which the hospital re-assigned the operating room slot to another surgeon, the patient was not fit to undergo surgery after all, or the surgery schedule was too busy for the elective surgery to take place.

The study team sought to assess and characterize the no-show events in ambulatory clinics and the non-utilized appointments for elective operations in a public hospital in Israel.

## Methods

The study was carried out between August 2016 and January 2017 in the ENT, Orthopedics and General Surgery Departments of the Jerusalem-based Shaare Zedek Medical Center (SZMC). These high-volume departments receive approximately 358,000 outpatient visits annually and operate on roughly 10,000 patients annually. The study team examined no-shows in the outpatient clinics and non-utilized appointments in the operating theater.

The first phase was conducted in the orthopedics and ENT services, both operating room and ambulatory care. The second phase included ambulatory care only in general surgery, ENT and orthopedics services.

The study was conducted using the hospital’s database in order to retrieve information regarding all visits and operations that were scheduled to occur in the six months between August 2016 and January 2017. The events categorized as no-shows were those outpatient appointments to which patients did not arrive as scheduled, leaving the appointment slot empty at the hospital’s outpatient clinics. The events categorized as non-utilized involved patients who were registered in the surgery schedule on a particular morning, but whose procedures did not take place on that particular day.

In the operating rooms, the documentation of the causes of non-utilized appointments cases was recorded at the end of each workday by staff. The study team collected these reports for analysis.

For missed ambulatory care visits, we contacted no-show patients chronologically and sequentially until 50 patients replied (25 in each clinic) by telephone. Responders were first asked their reason for not arriving at their scheduled appointments and they were then asked additional questions relating to their medical concerns, method of arrival, reminder status and level of satisfaction with the service (Appendix).

In the second phase, we reviewed a complete working week in Orthopedics, ENT and General Surgery ambulatory clinics, to asses all missed scheduled appointments and determined the type of no-show appointments according to the summary of each patients’ medical file.

It’s important to note, that there is no overbooking policy at the hospital’s ambulatory care clinics.

## Results

### Operating rooms

Following the examination of data in the operating rooms between the months of August and December 2016, the following results were found: Overall 3821 elective operations (all specialties) were scheduled to take place in the operating rooms. A total of 3608 elective surgical operations were performed, while 213 procedures (6%) that appeared in the morning schedule plan were not performed.

A total of 516 elective surgical procedures were planned in the ENT department, and 551 were planned in the Orthopedic department, of which 30 (6%) and 56 (10%) procedures did not take place (respectively). Cancelation causes are presented in Table 1.

**Table 1:** Causes of non-utilized surgical procedure appointments

	Orthopedics	ENT	All Operations
Scheduled	551	516	3821
Performed	495 (90%)	486 (94%)	3608 (94%)
Reason for cancelation			
Unknown reason	16 (28%)	6 (20%)	62 (29%)
Patient postponed	13 (23%)	4 (13%)	29 (14%)
Emergency surgery	6 (11%)		12 (6%)
An operation that extended for longer than planned	4 (7%)		6 (3%)
Patient medical problems	4 (7%)	10 (33%)	33 (16%)
Lack of medical equipment	2 (4%)		
Patient did not arrive at surgery and did not notify	2 (4%)	4 (13%)	11 (5%)
Incorrect summons due to an administrative error	2 (4%)		4 (2%)
Doctor cancelled appointment permanently	2 (4%)	2 (7%)	10 (5%)
Patient did not fast	2 (4%)		
Doctor postponed the appointment date	2 (4%)		4 (2%)
Schedule was too busy	1 (2%)	3 (10%)	28 (13%)
Patient cancelled appointment permanently		1 (3%)	
Other reasons			10 (5%)
No approval for surgery			4 (2%)
Total	56	30	213

Most frequently, there was no documentation for the reason of surgery cancellation. Of the reasons that were documented, the most common reasons were patient last-minute postponement (14%) and patient medical issues (16%) that prevented them from undergoing surgery.

### Ambulatory care clinics

In the ENT department, we examined the data regarding appointments that were scheduled between November 29th and December 7th, 2016. A total of 423 appointments were scheduled, 360 appointments took place on the scheduled date, and 63 patients did not reach the scheduled appointment (15%).

In order to reach 25 responses, we contacted by phone the first 47 listed patients in chronological order; 18 patients did not answer our phone calls and four did not agree to participate.

In the orthopedics department, we examined the data regarding appointments that were scheduled between December 1st and 7th, 2016. A total of 565 appointments were scheduled, 476 appointments took place on the scheduled date and 91 patients did not reach the scheduled appointment (16%). In order to reach 25 responses, we contacted by phone the first 68 listed patients in chronological order; 33 patients did not answer our phone calls and 10 did not agree to participate.

The overall main reason given by patients for non-utilized appointments in ENT and orthopedics departments was administrative issues (26%), one of which was that patients who actually arrive at the appointment but were registered as no-shows. The second most common reason was that the patient forgot the appointment (18%). Also notable is that in the orthopedics department almost a third of the patients did not arrive at their appointment due to acute illness that prevented them from arriving (Table 2).

**Table 2:** Causes of ambulatory care No-shows on telephone survey

	ENT *n* = 25	Orthopedics *n* = 25
Administrative problems	7 (28%)	6 (24%)
Forgot to attend	5 (20%)	4 (16%)
No longer needs appointment	4 (16%)	2 (8%)
Cancelled appointment	3 (12%)	
Sudden constraint	2 (8%)	1 (4%)
Did not receive guarantee from HMO	1 (4%)	2 (8%)
Tardiness	1 (4%)	1 (4%)
Parking	1 (4%)	
Illness of a relative	1 (4%)	
Illness		7 (28%)
Doctor did not arrive		1 (4%)
Religious festival		1 (4%)

* Administrative problems: An appointment was made at the wrong clinic, problems with registration in the hospital registry (a patient arrived and the system registered that he/she did not arrive, a patient received a phone call from the hospital about cancelling the appointment and instead registered that the patient did not arrive), the patient arrived but without the appropriate referral, the patient arrived without the appropriate tests, the patient was told that the appointment was scheduled without his/her prior knowledge, the patient arrived for the scheduled appointment but was referred to a private medical clinic

* No longer needs an appointment – the patient managed to find another solution for his/her medical issue and did not call to cancel the original appointment

* Cancelled appointment – the patient called to cancel the appointment but it registered as a No-show in the system

* Tardiness - the patient was late for the appointment and was not seen by anyone in the clinic

Table 3 describes the types of missed appointments as they appear in the patients’ medical records. The two common types of appointments missed in both departments, were medical follow-up (44%) and post-operation follow-up (42%).

**Table 3:** Type of No-show appointments

	ENT	Orthopedics	Total
Follow up medical care	9 (36%)	13 (52%)	22 (44%)
Post-Operation Follow up	12 (48%)	9 (36%)	21 (42%)
Pre-Operation	3 (12%)	3 (12%)	6 (12%)
Biopsy	1 (4%)		1 (2%)
Total	25	25	50

### Patient questionnaire

After information regarding the type of appointment and the cause for non-arrival were extracted from clinic records, patients were asked to answer the questionnaire (Appendix). Not all patients agreed to answer all of the questions in the questionnaire; the compliance rate varied according to the different question asked and ranged between 26% (13 patients) and 100% (all 50 patients).

A summary of the responses of the patients who answered the remaining questions are described as follows.

There were 17/43 patients (39%), five ENT patients (25%) and 12 (52%) orthopedics patients, that were concerned that their medical problem for which they had their appointment would recur.

Out of 50 patients who replied to the question – “did you find a solution to your medical issue”, 26% stated that they had found a medical solution in another location. Among them, 46% had found a solution in another clinic / with another doctor at SZMC. The rest, 54% found a solution in other clinics/hospitals, other than SZMC.

Out of 36 patients who replied to the question regarding the method of arrival, 64% arrived with their private vehicle, and the other 36% arrived via public transportation. Out of 34 patients who provided information about need of an escort, 53% stated they needed to be escorted while visiting the clinic. The majority (62%) of Orthopedics patients were in no need for an escort.

Out of 50 patients who replied to the question – “Did you receive a reminder for your appointment?”, 34% stated that they received a reminder for the appointment, 22% stated that they did not receive a reminder for the appointment, and 44% didn’t remember whether or not they received a reminder.

Out of 50 patients - 10% stated that they had never visited the clinic nor the hospital before.

Finally, out of 39 patients that replied the question - 77% stated they would recommend treatment at SZMC to a friend/family member.

While giving the opportunity for further comments, a sporadic number of patients mentioned that they had attempted to inform the clinic in advance that they were missing their scheduled appointments, but they did not succeed. The main reasons given for this were: inadequate automated call-back services, the lack of an answer service at a specific clinic with an option of automatic rescheduling, and lastly, the lack of a customer service representative to cancel or postpone the appointment.

### Analysis of patient files for no-show clinic appointments

In the second phase of the study we examined the patient files in general surgery, orthopedics and ENT.

In the general surgery clinic, 415 appointments were scheduled to take place during the week of January 1st to 7th, 2017. However only 338 appointments took place and 77 patients (19%) did not arrive for their scheduled appointments. Patients records were reviewed for all no shows.

In the orthopedic clinic, 566 appointments were scheduled to take place during the week of December 7th to 14th, 2016, but only 462 appointments took place. One hundred four patients (18%) did not arrive for their scheduled appointments and patient records were reviewed for 70 of these patients.

In the ENT clinic, 367 appointments were scheduled to take place during the week of December 7th to 14th, 2016, but only 313 appointments took place. Fifty four patients (15%) did not arrive for their scheduled appointments. Records were reviewed for all 54 of them.

The distribution of the type of appointment, as documented in the medical records, is presented in Table 4. For many of the appointment, the type of appointment could not be determined from medical records. Among the appointments with a clear purpose, the most common appointments missed were post-operation follow-up.

**Table 4:** Types of appointments missed extracted from medical records

	ENT	Orthopedics	General Surgery
Unknown reason	21 (39%)	16(23%)	18 (23%)
Follow up of chronic condition	8 (15%)	18(26%)	15 (19%)
Pre-surgery	3 (5%)	8 (11%)	9 (12%)
Visit after hospitalization	5 (9%)	1 (1%)	4 (5%)
Review after injury	1 (2%)	4 (6%)	
Review after fracture		5 (7%)	
Post-surgical follow-up			
First follow up after surgical operation	5 (9%)	1 (1%)	16 (21%)
Second follow up after surgical operation	7 (13%)	12 (17%)	11 (14%)
Third follow up after surgical operation	3 (5%)	4 (6%)	3 (4%)
Fourth follow up after surgical operation	1 (2%)	1 (1%)	2 (3%)
Second or further follow up after surgical operation	11 (20%)	17 (24%)	16 (21%)

## Discussion

Our study results indicated that in SZMC, the extent of non-utilized appointments is lower than reported in the literature on operating rooms (6%).

The rate of no-show phenomenon in the clinics is similar to that reported in the literature (15 to 19%) [8, 9].

Some interviewed patients revealed that the process of cancelling an appointment by the patient was difficult and sometimes generated a no-show case because the patient couldn’t reach an operator to cancel the scheduled appointment successfully. In addition, an important finding in our study was that there were several patients who arrived at their scheduled appointments and the appointment took place, but due to a system registration error, it was registered as a no-show case.

Interestingly, the results of the telephone questionnaire show that most of the patients who did not arrive at the clinic were satisfied with the service they received at the SZMC and would recommend this hospital to a friend/relative. This suggests that the majority of no-show events were not due to dissatisfaction.

We did not measure unused OR (operating room) time during workdays and cannot report whether non-utilization resulted in reduced efficiency of the OR. There are no available comparative data from other medical centers in Israel from which we can learn whether the surgery cancellation rates reflect hospital efficiency or lack of efficiency. Focused assessment and methodology are required in order to develop strategies to cope with surgery cancellation.

We note that the reason for approximately 30% of all cancelled surgeries was unknown. This indicates that there is a weakness in the documentation process and this limits the ability of the hospital to cope with the phenomena. As mentioned previously, ambulatory clinic records are also not always correct. Improving the documentation process, as well as training the staff to record the reason for the cancellation of an operation, are important elements for optimal management.

The outpatient clinics’ no-show rates are in the lower range of what has been published in the literature (10–30% [4]). Still, in terms of financial cost and exploitation of resources, they nonetheless translate into an immense amount of waste. A Canadian study estimated that the average cost per missed visit to the hospital was $95 and that the total cost to the economy was estimated at $211 per missed visit [10]. Assessing the economic efficiency of a medical service requires additional data regarding throughput – we can have few scheduled appointments with low cancellation rates or many scheduled appointments with higher cancelation rates, but with an overall provision of more medical care to more patients per day.

The search for a solution to reduce no-show rates requires us to face the particular reasons for which the patients did not arrive. Facing forgetfulness by patient-reminders through various forms (post, telephone, text messages, or e-mail) for a scheduled appointment have proven to significantly lower the frequency of no-show cases. However, it has not been established which type of reminder is the most effective [11]. A positive outcome of appointment reminders is an increase in the rate of initiated cancellations by patients who did not intend to arrive at the appointment [12]. This would be particularly relevant at SZMC since in the telephone questionnaire we conducted, patients noted that they tried to cancel the appointment and couldn’t reach the calling center.

A widespread method of dealing with the no-show phenomenon is deliberately overbooking appointments [13]. Overbooking can significantly increase clinic service volume by increasing the availability for patients and the overall productivity of the clinic, which leads to a reduction in costs and improved patient satisfaction. In contrast, overbooking can prolong queues in clinics and increase patient dissatisfaction prior to the appointment in hand. As mentioned before, SZMC does not currently have an overbooking policy.

Another method of coping with the no-show phenomenon is the use of probability models that consider a patient’s personal data, such as social characteristics, gender, previous no-show cases, in order to predict the likelihood of future no-show cases [13, 14]. Coupling this with reminders to these “high-risk” might prove useful.

One can suggest lowering the no-show rates by introducing fines or penalties when receiving treatment after not arriving for the scheduled appointment, for example transferring the patient to the end of the queue. There is limited empiric literature on no-show penalties, various ethical dilemmas, and unclear profit and productivity consequences [15]. Additionally, penalties are illegal in Israel because they lead to inequality in healthcare provision [16].

The computer information systems and staff documentation in SZMC should be improved. With valid data, the system could locate patients with higher risk factors for no-show cases, particularly those who have already had a no-show case in the past, as described by others [2]. This will also help lower false no-show/non-utilized appointments (appointments that were registered as no-shows but which actually took place).

An important finding in our research is that many of the appointments that did not take place were post-operative follow-up appointments (32% of all causes for no-show cases and 44% of known causes). Perhaps there is a need for a closer consideration of the importance of these appointments, particularly beyond the first post-operative follow-up. In many cases, the post-surgery follow-up appointments are crucial for monitoring clinical improvements, including wound healing, rehabilitation, and so on. However, in some cases, surgery is the definitive solution to the problem and therefore patients who no longer suffer from the original problem do not feel the need to attend the scheduled post-surgery appointment, particularly when the appointment was scheduled a long time in advance. By judiciously removing unnecessary appointments from the appointment schedule, it should be possible to significantly shorten waiting periods for patients and leave regular intervals for receiving patients after surgery who need medical treatment. We suggest that beyond the first post-operation visit – surgeons need to provide the rationale for additional visits – this would limit the frequency of appointments which are automatically proposed without attention to their necessity. However, we do not have the overall rate of such appointments in the surgical clinics, and our conclusion should be considered with that limitation in mind. Modern telemedicine tools can present alternative strategies for post-operative surveillance [10].

### Study limitations and directions for further study

This study was performed in a public health system with unique incentives and reimbursement system; therefor its conclusions should be generalized to other health systems only with a good deal of caution. Indeed, generalizations even to other Israeli hospitals should also be undertaken cautiously, as the patients at SZMC could well be different from patients at other hospitals, and hospitals may differ in how they address the issue of no-shows. Unfortunately, at present we do not have comparative data from other Israeli hospitals.

We note that the data from the appointments that did take place was not examined. In future studies, this data should be compared to data from the appointments that did take place, in order to assess whether there are significant differences between patients who kept their appointments and those who did not.

A possible limitation of the study is the lack of patient no-show history and their socio-economic information. This information, along with personal characteristics such as age and gender, might help future studies identify ‘risk factors’ for no-show patients. Comparing these data to patients who do arrive could improve our knowledge about intervention options. However, we feel that caution should direct any implementation of institutional strategy according the personal characteristics; this could raise ethical concerns and might not be appreciated by the public.

The study period included the Israeli holiday period, in which people are probably more prone to appointment cancellations. Future studies can examine longer time periods that do not include the specific Israeli holiday period. We should also note that using only one week can be sensitive to one-time phenomena, and longer observation periods might be more informative, though not necessarily.

Lastly, regarding the telephone survey, it’s important to note the low response rate and low number of participants. Also, as in all volunteer-based telephone surveys, there could be a selection bias due to personality characteristic of participants.

Based on this preliminary study, future studies could explore the possibilities of interventions in reducing no-show and surgery cancellation rate.

## Conclusions

In our exploratory research, we demonstrated that the rates of operation non-utilization in ENT and Orthopedics departments in SZMC were lower than those documented in the literature, and the rates of no-show events at the ambulatory clinics are similar to rates in the lower range of rates reported in the literature.

In order to reduce no-show rates, we suggest careful consideration of the necessity of post-operative appointments, since these appointments are common among no-show events and possess the lowest risk for clinical damage.

The findings presented can help identify appointments with a higher probability of no-show, and can aid appointment management, reducing the allocation of resources to appointments with a high probability of low utility, improve the quality and performance at hospitals, and, more importantly, improve the provision of medical services and the health of our patients.

## Data Availability

The datasets generated and/or analyzed during the current study are not publicly available due to patient confidentiality but are available from the corresponding author on request.

## Appendix

### Phone questionnaire with no-show patients

1. Hello, we’re holding a study regarding the service given in ambulatory clinics in SZMC. It appears in our records that you didn’t arrive at your scheduled appointment in … clinic on the date ….
  May I enquire the reason for your non-arrival?
2. Did you find a solution for your medical issue? If yes, where?
3. Are you concerned regarding recurrence of the medical issue?
4. Did you visit SZMC before?
5. What is the method of arrival planned for this visit?
6. Do you need an escort for visiting the clinic?
7. Did you receive a reminder for the planned appointment?
8. Would you recommend treatment at SZMC to a friend/family member?
9. Is there anything else you would like to mention regarding this visit or the service you are receiving at the clinic?
10. Thank you for your cooperation.

## Abbreviations

ENT: Otorhinolaryngology
OR: Operating room
SZMC: Shaare Zedek Medical Center
